# Progress in the clinical application of constraint-induced therapy following stroke since 2014

**DOI:** 10.3389/fneur.2023.1170420

**Published:** 2023-05-19

**Authors:** Yinxing Cui, Ningyi Ma, Xuncan Liu, Yawen Lian, Yinghua Li, Guoxing Xu, Jiaming Zhang, Zhenlan Li

**Affiliations:** Rehabilitation Medicine Department, First Hospital of Jilin University, Changchun, China

**Keywords:** stroke, CI therapy, rehabilitation, motor dysfunction, aphasia

## Abstract

Stroke is a group of cerebrovascular diseases with high prevalence and mortality rate. Stroke can induce many impairments, including motor and cognitive dysfunction, aphasia/dysarthria, dysphagia, and mood disorders, which may reduce the quality of life among the patients. Constraint-induced therapy has been proven to be an effective treatment method for stroke rehabilitation. It has been widely used in the recovery of limb motor dysfunction, aphasia, and other impairment like unilateral neglect after stroke. In recent years, constraint-induced therapy can also combine with telehealth and home rehabilitation. In addition, constraint-induced therapy produces significant neuroplastic changes in the central nervous system. Functional magnetic resonance imaging, diffusion tensor imaging, and other imaging/electrophysiology methods have been used to clarify the mechanism and neuroplasticity. However, constraint-induced therapy has some limitations. It can only be used under certain conditions, and the treatment time and effectiveness are controversial. Further research is needed to clarify the mechanism and effectiveness of CI therapy.

## 1. Introduction

Stroke is a group of cerebrovascular diseases with high prevalence and mortality rate (1). In the United States, about 795,000 people experience a new or recurrent stroke each year, and the overall stroke prevalence among Americans over 20 years of age is estimated at 3.3% (2). Globally, the prevalence of stroke was 89.13 million, and 7.08 million deaths can be attributed to stroke annually (2). Stroke is the second leading cause of death worldwide, with an estimated 5.5 million mortality rate annually, and its high morbidity results in long-term disability in up to 50% of survivors (1). Stroke can induce many impairments, including motor and cognitive dysfunction, aphasia/dysarthria, dysphagia, and mood disorders, which may reduce the quality of life (QoL) among the patients (3–5).

Constraint-induced (CI) therapy has been proven to be an effective treatment method for stroke rehabilitation. It was originally used for the rehabilitation of upper limb motor function, and gradually expanded to that of lower limb motor function recovery and aphasia treatment (6–10). Among them, constraint-induced movement therapy (CIMT) is a rehabilitation treatment method based on neuroscience and primate behavior research, which is effective in the rehabilitation of motor function after stroke (10). Constraint-induced aphasia therapy (CIAT), also known as intensive language-action therapy (ILAT), is an effective treatment for aphasia (11).

**Figure 1:**
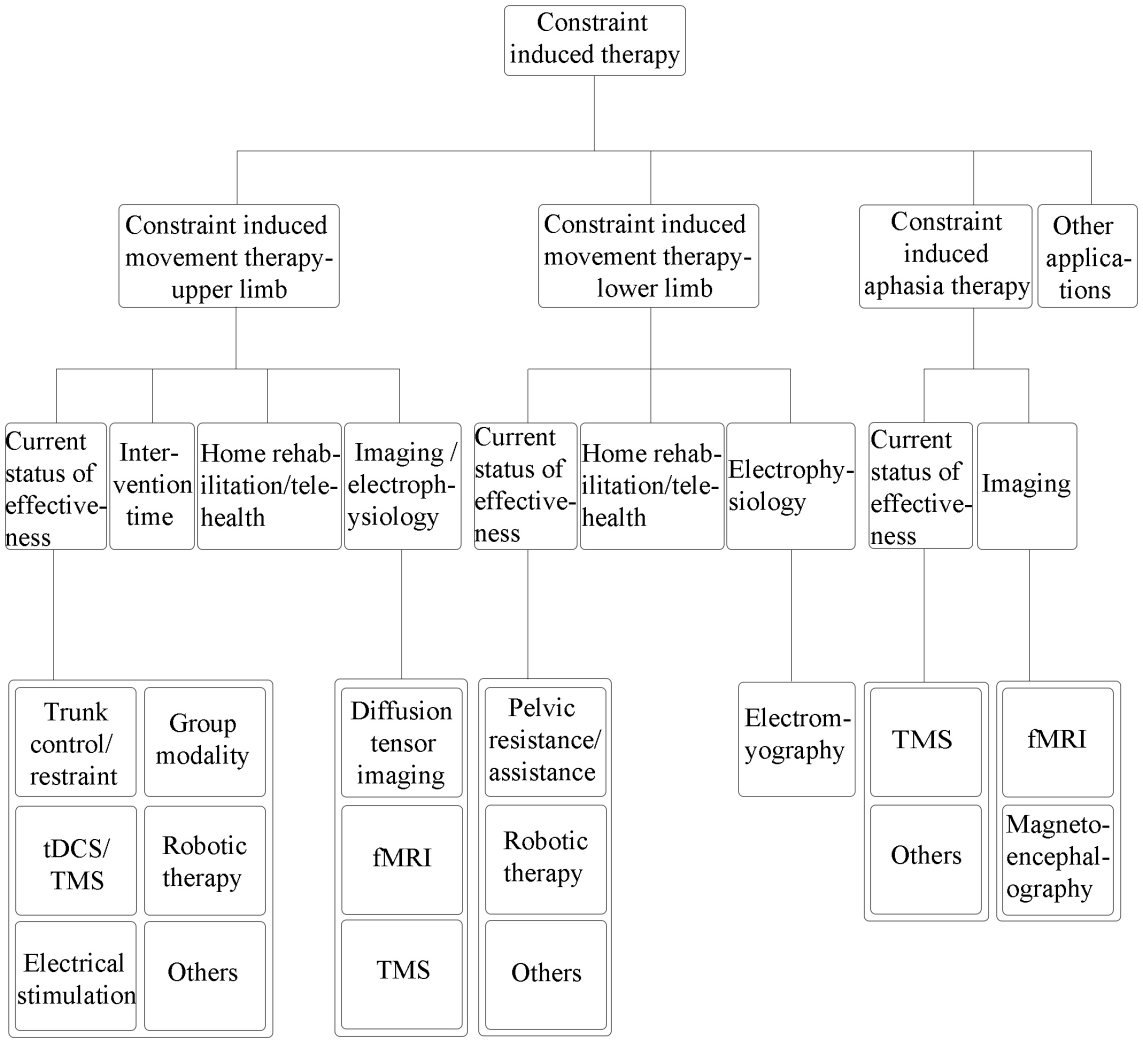
General writing framework of the article.

A literature search was conducted based on a selective search in the PubMed/MEDLINE databases to search the literature from 2014 up to 2022. We used search terms related to “stroke,” “constraint-induced movement therapy,” “intensive language-action therapy”, “constraint-induced aphasia therapy,” and “constraint-induced therapy,” to review the clinical application of CI therapy in upper and lower limb motor dysfunction, aphasia, and other impairments after stroke. The literature search was limited to articles published in English, and the full text was available. We mainly included clinical studies on adults and excluded studies that the CI therapy was not an important method. The general writing framework was shown in Figure 1.

## 2. Clinical study of CI therapy in the treatment of upper limb

**Figure 2:**
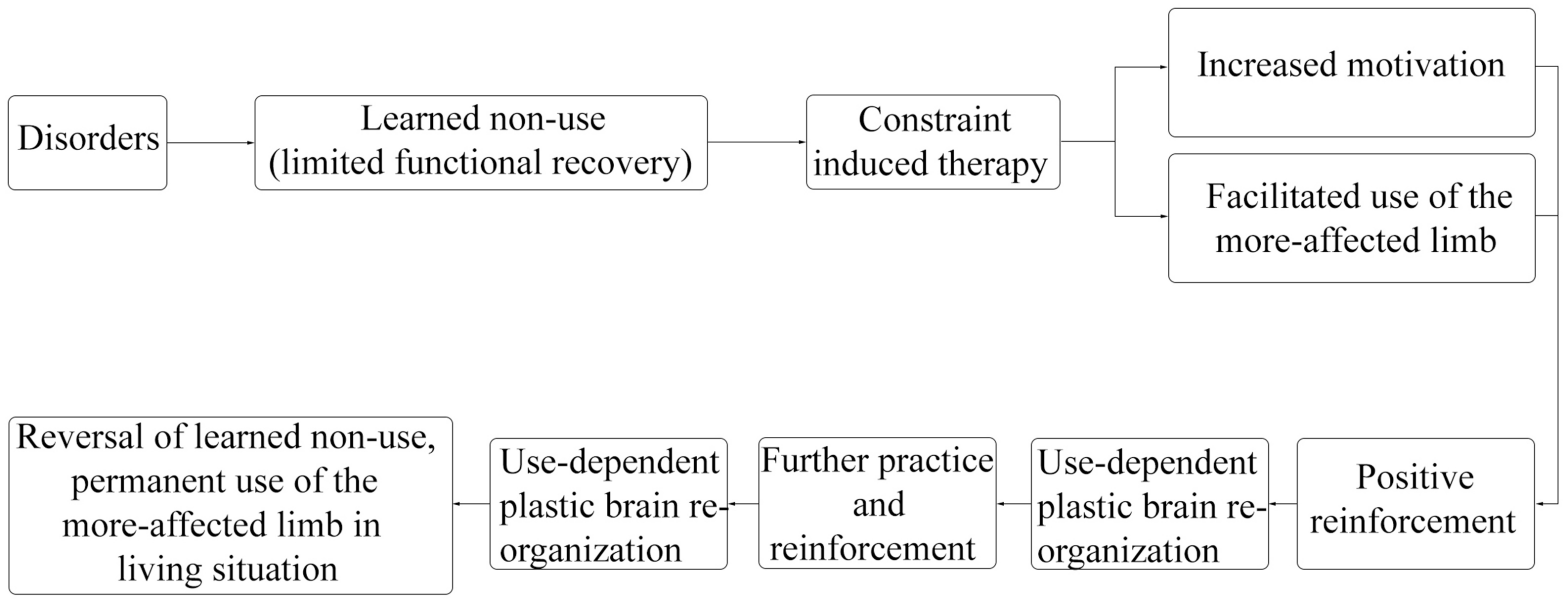
Recovery process of the CI therapy for upper limp.

Upper limb motor dysfunction after stroke is a common complication, about 65% of patients were unable to engage the affected hand in daily activities 6 months after stroke (12). The CIMT for upper limb program consists of three components: ① repetitive, task-oriented training of the more-affected limb (shaping, and task practice), ② application of the “transfer package” of adherence-enhancing behavioral strategies that facilitate the transfer of therapeutic outcomes from the treatment setting to daily living situations (such as home diary, problem solving, and behavioral contract), and ③ constraining use of the more-affected limb, sometimes by restraining the less-affected limb (13, 14). CIMT applies movement techniques, behavioral techniques, and restriction methods to increase the frequency of using the more-affected limb by limiting the use of the less-affected limb, improve the quality of movement of the more-affected limb in real life, prevent or correct learned non-use, and ultimately improve the motor function of the more-affected limb (15). The efficacy of CIMT is considered to be associated with changes in brain plasticity (10, 16). The recovery process of CIMT was shown in Figure 2 (10, 13–16). The traditional CIMT approach requires restraint of the less-affected limb for 90% of the waking time and about 6 hours of training each weekday for 2 weeks (17). However, it was difficult to implement clinically; in addition, patients and therapists reported difficulties applying this approach (18). Therefore, the modified CIMT (mCIMT) has been created. While compared to the traditional CIMT, evidence suggested that mCIMT had similar functional recovery of the affected limb (18, 19).

### 2.1. Current status of effectiveness

The effectiveness of CIMT can be considered through various rehabilitation evaluation scales. Some studies have found improvements in motor function and affected limb use after CIMT intervention compared to pre-treatment (20, 21). Some studies comparing conventional rehabilitation therapy with CIMT (or CIMT combined with conventional rehabilitation), the results had shown improvements in motor function, spasticity, and occupational performance with CIMT (or CIMT combined with conventional rehabilitation) (22–26). In addition, the improvement of upper limb function could be related to the performance of activities of daily living (ADL) with CIMT (20), and CIMT might improve depressive symptoms (21).

Some studies compared CIMT with other therapies. A study showed significant improvement in spasticity and upper limb motor function in the mCIMT group compared with the proprioceptive based training group (18). Another study showed significant improvements in motor function and ADL in the CIMT group compared with the unconstraint task-oriented training group (27). However, some studies had shown that CIMT did not significantly improve function in patients compared to other therapies (28, 29), but the CIMT did not increase fatigue (28). Considering that CIMT is superior to some, but not all of the therapies; the best treatment method for the patient needs to be selected according to the condition and needs.

CIMT can be combined with other therapies. A study combined mCIMT with virtual reality training, the upper limb motor function improved (30). Another study used mCIMT or intensive conventional rehabilitation based on botulinum-A toxin injection, the results showed that the motor function and ADL of botulinum-A toxin injection combined with the mCIMT group were significantly improved between groups (31). In addition, a study combined short-term CIMT with visual biofeedback training, grasp and pad pinch function improved significantly compared to the conventional occupational therapy group (32). Yoon et al. combined mirror therapy with CIMT, and the results showed that the improvement of upper limb motor function was more significant than that of CIMT alone (33). It can be considered that CIMT combined with other treatment methods may have additional effects.

Trunk compensatory strategies may hinder long-term functional recovery of the upper limb (34). CIMT program could add trunk restraint. Some studies have demonstrated that CIMT combined with trunk restraint is significantly superior to CIMT alone in terms of upper limb motor function, ADL, and the use of the hemiplegic upper limb (35–37). In addition, another study combined auditory feedback for trunk control with mCIMT, the results suggested the upper limb function improved significantly compared to mCIMT alone (34). However, a study suggested that adding trunk restraint to mCIMT had no additional benefit (38). The effectiveness and mechanism of trunk control require further research.

Sometimes group modality may provide a foundation for communication and emotional support, stimulate the healing process, and transfer these improvements into functional independence and participation in daily activities (39). Some studies comparing group and individual modalities of CIMT indicated that motor function, amount of upper limb use, and functional independence increased more significantly in the group modality (39, 40). In addition, a study had shown that the amount of upper limb use daily significantly improved with group-based CIMT (41). The group modality can improve communication between patients, and may save the treatment time of the therapists to provide recovery for more patients; further research is needed to find out the most suitable group modality therapy for clinical use.

Some studies have combined transcranial direct current stimulation (tDCS) or transcranial magnetic stimulation (TMS) with CIMT. Two studies showed that tDCS combined with mCIMT group improved upper limb motor function compared with mCIMT alone group (42, 43). However, another study suggested that tDCS combined with mCIMT just improved the amount of the paraplegic upper limb use, with no statistical difference in motor function compared to mCIMT (44). In addition, one study combined CIMT with different tDCS (anodal stimulation in ipsilesional primary motor cortex versus anodal stimulation in ipsilesional premotor cortex versus sham stimulation), the motor function and ADL were improved and the muscle tone was decreased significantly in the premotor cortex group compared with the other two groups (45). Moreover, a study combining dual tDCS and peripheral neuromuscular electrical stimulation in a treatment group based on CIMT found significant improvements in motor function and use of the paralyzed upper limb compared with CIMT alone (46). Another study combining low-frequency repetitive TMS (rTMS) with intensive occupational therapy showed significant improvement in motor function compared to CIMT (47). tDCS/TMS has been widely used and can be combined with CIMT. However, it may not be ruled out that other therapies combined with tDCS/TMS are more effective. Moreover, different brain regions have different gains from stimulation, so further research is needed to find the most suitable method.

Some studies have involved robotic therapy. A study compared robot-assisted therapy to CIMT, and the results suggested that both of them could improve the function of the patients, but there was no significant difference in motor function between groups (48). Some studies that added CI therapy to robot-assisted therapy showed a reduction in compensatory trunk movement during the task, with more significant improvements in motor function and ADL compared with robot-assisted therapy alone (49, 50). Robotic-based rehabilitation is a hot research field, and the studies have shown that it can be combined with CI therapy. Robot-based therapy can provide precise control and real-time monitoring of the patients; through further research, we may find out suitable treatment methods for patients and achieve precise rehabilitation.

Electrical stimulation is also a common method in rehabilitation. A study combining modified CI therapy and peripheral nerve stimulation suggested that the improvement of motor function was more significant than that of modified CI therapy alone (51). Another study used electromyography-triggered neuromuscular stimulation on the patient with an unfavorable prognosis, the result suggested that there was no significant difference compared to usual care (22). It is considered that electrical stimulation may have an additional effect in patients eligible for CIMT, but in patients with low function, it may not possible to improve function by electrical stimulation alone.

In addition to rehabilitation methods, patient management is also essential. Self-regulation is designed to improve patients’ self-awareness and assist in identifying their functional problems, thus facilitating the recovery process (52). A study combining self-regulation and mCIMT has found additional effects on functional recovery in patients after stroke (52). In addition, a study used the mobile health platform to help patients improve self-management and timely communication. The study combined CIMT with the use of mobile health platform, the results showed significant improvements in motor function and ADL compared to conventional rehabilitation program (53).

### 2.2. Discussion on the intervention time

Several studies have compared early and late delivery of CIMT. A study showed that the early applied CIMT group had greater improvement in motor function than the late applied group (54). But another study found that early delivery of CIMT was as good as late intervention; however, the early CIMT intervention group showed a faster recovery curve than the late intervention group (55). Considering that according to the patient’s condition, CIMT can be intervened early; if CI therapy has not been used in the early stage, it can also be added when necessary, and there is no need to worry about the late intervention of CIMT may be ineffective.

### 2.3. Discussion on the home rehabilitation and telehealth

Home rehabilitation and telehealth are current research hotspots. In the post-coronavirus disease 2019 era, the development of home rehabilitation is particularly important. CI therapy can also be combined with telehealth and home rehabilitation, and gradually applied to out-of-hospital care. With telehealth, treatment services can provide to individuals who may not be able to visit clinics, and there is an internet-based CIMT, which is the mCIMT approach combined with telehealth (56). In addition, telehealth can take the approach of games, and its efficacy can be similar to that of in-clinic rehabilitation (57). And home rehabilitation also can be via an in-home game program (58).

A study using mCIMT in combination with telehealth in high/low functioning patients showed significant improvements in motor and ADL functions in both groups (56). Another study showed the telehealth CIMT group was no worse than the face-to-face CIMT group in using the more-affected upper limb (59). In addition, a study comparing in-home CIMT with conventional rehabilitation found that in-home CIMT could improve the use of the affected upper limb more effectively than conventional therapy, but was not superior in motor function improvement (60). However, the results were controversial. Another study showed that there was no significant difference between in-home CIMT and conventional rehabilitation in motor function and participation (29). Furthermore, a study analyzed factors associated with QoL in different CIMT programs (in person/in home), and found that improvement in QoL was associated with gains in upper-limb use but not with motor function (61). Considering that telehealth and home rehabilitation combined with CIMT are useful for stroke patients who need long-term rehabilitation, but may need to improve treatment protocol to ensure the efficacy; further research can be done to develop more programs to provide convenience for patients.

### 2.4. Imaging and electrophysiology – discussion on the mechanism

Diffusion tensor imaging can be utilized to show the effects of CIMT on the fibers of the corticospinal tract (CST). A study showed that patients with disrupted or displaced CST had lower motor function in pre-treatment than those with unaltered CST. However, this had no significant difference in their ability to benefit from CI therapy (62). Another study showed similar results. The integrity of CST correlated with patients’ motor function, and there was no significant difference between left and right hemiplegia. The post-treatment benefit was not associated with CST integrity or lesion volume (63).

Some studies applied functional magnetic resonance imaging (fMRI). A study showed the CIMT led to increased activity of the lesioned hemisphere dorsal premotor cortex compared to the untreated group, and no changes in laterality index were observed in the primary motor cortex (64). Upper limb dominance did not affect the improvement of upper limb function after CIMT (65). However, fMRI showed that the images were different according to the dominant/non-dominant side of the upper limb paralysis. When less-affected upper limb constraint was added to the right-handed left/right hemisphere stroke patients during the paralyzed arm elevation, the study had shown that in right hemisphere stroke patients, it led to the silence of contralesional cortical areas while maintaining ipsilesional activation of the sensorimotor cortex. And in patients with left hemisphere stroke, the same situation led to bilateral reduction of cortical activation (66). Considering that further studies on hand dominance are needed, it is also possible that left/right hemisphere stroke has different functional mechanisms.

TMS can be used for electrophysiological evaluation. Motor-evoked potentials (MEPs) were significantly improved, and the ipsilesional silent period declined in the mCIMT group compared to baseline in acute stroke (67). However, there were no long-term differences in motor function or electrophysiological parameters between mCIMT and standard therapy groups (67). Another study showed that when compared pre-and post-treatment MEPs, there were no significant improvements in resting motor threshold, central motor conduction time, and amplitude with conventional rehabilitation; and the MEP parameters in CIMT group were significantly improved (26). In addition, acute stroke could cause the interhemispheric excitability imbalance. It is considered that tDCS can reduce the interhemispheric excitability imbalance through the change of MEP (68). Furthermore, a study compared early and late applied CIMT, the results showed that greater cortical recombination occurred in the late group in terms of map size and position, considering that the recovery mechanism may have changed over time (54).

Imaging and electrophysiology are important methods to clarify the mechanisms that reflect neuroplasticity changes in CIMT and other recovery methods. At present, much of the latest research in this field tends to be animal experiments (69), and more human studies are needed. It is also important to edit appropriate functional tasks in fMRI.

## 3. Clinical study of CI therapy in the treatment of lower limb

Lower limb motor dysfunction after stroke is also a common disorder. There are up to 35% of stroke patients with initial lower-limb paralysis who do not regain physical function, and 20–25% of them are unable to walk without full physical assistance (12). CIMT for the lower limb was modified from the original upper limb CIMT (14). CI therapy for lower limb has been widely used in neurological disorders (10).

### 3.1. Current status of effectiveness

In CIMT for lower limb, the number of practice tasks, rather than practice time, may play an important role in functional recovery. Therefore, it may be more convenient to use the CIMT protocol with the number of repetitions than the CIMT protocol with the number of practice hours, so the mCIMT scheme is preferable (70). In addition, a study found improvements in the use and function of the paralyzed lower limb after CIMT (71). Another study showed that CIMT significantly improved patients’ balance function, lower limb motor function, and walking speed compared to conventional rehabilitation, and many of these improvements were sustained after 3 months (72).

Lower limb restraint can be accomplished with “pelvic resistance/assistance.” In the “pelvic resistance” condition, the lateral weight shift to the paralyzed side was improved compared to the “pelvic assistance” condition, while the “pelvic resistance” also improved overground gait speed and standing phase symmetry (73). Park et al. suggested that lower limb muscle activity, weight shift toward the affected side, and overground walking speed were significantly improved in the condition of a gradual increase of “pelvic assistance” force, which was better than that under the sudden increase. It was considered that the “gradual increase” during constrained induced walking might improve weight shift and enhance forced use of the paralyzed lower limb (74). Another study showed that pelvic resistance in the standing stage showed a greater increase in hamstring muscle activity in the paretic lower limb and improvement of the step length symmetry compared with constant resistance applied throughout the gait cycle (75). Restraint devices were omitted in lower limb CIMT, mainly due to safety issues like high risk of falls; meanwhile, wearing the device during the intervention led to unnatural gait and postural patterns (14). Resistance applied to the pelvis in the above studies provided a similar restraint effect to upper limb CIMT, which may have an additional effect on functional improvement.

CIMT for lower limb can also involve robotic therapy. A study used a rehabilitation robot to apply resistance to the less-affected lower limb, while assistance was applied to the more-affected side, and performed gait analysis. The results suggested that “Lokomat® constraint gait training” might improve knee flexion of the paralyzed lower limb, and the effect was better than that of conventional robotic gait training (76). The robot-assisted system can provide precise force changes. It can provide appropriate assistance and resistance force, may solve the problem of traditional lower limb CIMT with no resistance, and can be considered for clinical promotion.

### 3.2. Discussion on the home rehabilitation and telehealth

Home rehabilitation can also be used in lower limb motor dysfunction, and can adopt the game mode. One study compared game-based CIMT with game-based training, the results suggested that both of game-based CIMT and training could improve static and dynamic balance, and the CIMT had a greater effect on static balance control (77).

### 3.3. Electrophysiology – evaluation with electromyography

Studies related to lower limb CI therapy can be conducted by electromyography. Some studies have compared whether forcible use of the paralyzed lower limb by restraining the non-paralyzed lower limb, could improve the functional ability of the patients. One study showed that step length symmetry could be significantly improved during treadmill walking with constraints (78). Furthermore, during the stance phase, the electromyography of some lower limb muscles increased significantly at the early adaptation period. Meanwhile, under the constraint condition, the retention during the post-adaptation period was significantly greater than that under the treadmill-only condition (78). Another study has shown that applying controlled resistance force to the non-paralytic lower limb during early swing phase increased the use of paralytic lower limb and improved spatiotemporal symmetry of gait (79). In addition, compared to baseline, significant electromyography increases were observed in some muscles of the paralyzed lower limb when resistance force was applied during the early swing phase, and 30% maximum voluntary contraction resistance caused the highest level of muscle activity compared to 10% or 20% maximum voluntary contraction resistance (79). Studies on electrophysiology and imaging of lower limb CIMT are less common than those of upper limb CIMT, so more relevant studies should be conducted to help clarify the mechanism and neuroplasticity of CIMT for lower limb.

## 4. Clinical study of CI therapy in the treatment of aphasia

Aphasia is an acquired language disorder that affects all aspects of language-based communication: comprehension of speech, reading, writing, and speaking, and is a common disorder that affects the QoL among stroke patients (80). Aphasia treatment may require extensive training, behavioral and communicative relevance of the interaction during treatment, and should focus on the patients’ communication needs and possibilities. These principles have been adopted by a new treatment method called CIAT (81).

### 4.1. Current status of effectiveness

CIAT can be used in the treatment of aphasia after acute stroke (82). Meanwhile, patients with chronic aphasia for more than 1 year can also expect language improvement through CIAT (83). The benefits gained by CIAT may sustain long after treatment ended (84).

Studies have found that CIAT could improve the subjective language abilities (85) and depressive symptoms (86). Stahl et al. comparing CIAT and naming therapy suggested that CIAT significantly improved language performance, independent of the duration of the intervention (87). In addition, a study compared CIAT with multimodality aphasia therapy (M-MAT) and usual care, showed CIAT and M-MAT were better than usual care for aphasia. After intervention, M-MAT was beneficial for patients with severe aphasia, CIAT was beneficial for patients with moderate aphasia, but there was no difference between them for patients with mild aphasia (80). Through these studies, CIAT can be considered to improve aphasia, but different levels of aphasia may need different treatment methods. Furthermore, one study compared the efficacy difference between high-intensity and moderate-intensity CIATs. The results indicated that there was no statistical significance between them (88). It was considered that only increasing the daily treatment time might not gain additional effect.

Some studies showed CIAT could combine with rTMS. A study combined rTMS with CIAT, and compared to CIAT, found no additional effect of rTMS (89). Nevertheless, previous studies have found that both of CIAT and CIAT combined with rTMS could improve naming (90, 91). The results of the studies may be related to the small sample size, and further research is needed to determine whether it is necessary to combine rTMS based on CIAT.

### 4.2. Imaging – discussion on the mechanism

Functional imaging was used for the studies. Some studies used intermittent theta burst stimulation and CIAT. The language measurements suggested that intermittent theta burst stimulation combined with mCIAT indicated improvements, and these improvements were correlated with changes in the blood oxygenation-level-dependent (BOLD) fMRI in left inferior parietal lobe and right inferior frontal gyrus (92) or in right postcentral gyrus and bilateral supplementary motor area (93). Some other studies also have shown that the BOLD signal changes with fMRI correlated with improvement in the clinical aphasia test after CIAT (11, 94). However, the connection between aphasia therapy and neuroplasticity changes is controversial. One study found that language ability improved in stroke patients with CIAT, but the changed fMRI areas were mainly related to behavioral performance. Considering that language-related cortical plasticity may not have a specific effect on CIAT (95). A study used magnetoencephalography to suggest that CIAT improved language skills in patients, and language recovery was associated with changes of neuroplasticity in both cerebral hemispheres (96). CIAT was considered to be associated with language-related changes in neuroplasticity, but the results have not been consistent. Further studies on functional imaging are needed to clarify the mechanism of CIAT.

## 5. Other applications

Unilateral spatial neglect is a common consequence of stroke survivors, most of which occur after right hemisphere stroke, resulting in neglect of the left visual hemifield (97). One of the main reasons why CI therapy is effective is that it overcomes the learned non-use, and considering that CI therapy as an approach to treating unilateral spatial neglect is worth trying (97). Sleep may interfere with functional recovery. A study has shown that circadian preference and sleep quality affects the functional improvement among stroke patients after CI therapy (98).

## 6. Current problems

However, CI therapy has some limitations. It can only be used under certain conditions and cannot cover all patients. Patients who can use CIMT for upper limb always require certain motor functions, such as the active extension of the thumb and two or more fingers (≥10°) of the affected hand (22). Moreover, the treatment time and effectiveness are also controversial. The traditional CIMT including restraint of the less-affected upper limb to facilitate the use of the more-affected limb during 90% of the waking time (17), which was difficult to implement clinically. However, a study has shown no significant correlation between constraining time and functional outcomes (99). The necessity and the appropriate time of constraint need further study. In addition, a study has shown that CIMT improved motor function immediately after treatment, but no significant effect was observed after 6 months compared to conventional therapy (100).

## 7. Conclusion

CI therapy is widely used in stroke rehabilitation, and different CI therapies can meet diverse needs for recovery of patients. It can be combined with other treatment methods to provide additional benefits. Many studies have now demonstrated the effectiveness of CIMT not only by rehabilitation evaluation scales but also by electrophysiological and imaging methods. With the development of technology, CI therapy is linked to telerehabilitation, responding to the needs of patients with chronic dysfunction and/or inconvenient access to the clinic. However, CI therapy has some limitations. Requirements on basic motor function may limit its application in some patients. In addition, limiting the use of the non-paralyzed limb may affect the patient’s ADL and cause inconvenience. Moreover, its long-term effects need to be further studied. Meanwhile, further research is also needed to clarify the mechanism and effectiveness of CI therapy. In addition, the protocol needs to be improved. Furthermore, it needs to be integrated with modern technology and applied in clinical practice.

## Author contributions

YC drafted and revised the manuscript. NM made figures and helped in revision. XL and YLia performed the literature search and extracted the articles. YLi, GX, and JZ helped in writing and editing. ZL conceived and designed the manuscript. All authors contributed to the article and approved the submitted version.

## Conflict of interest

The authors declare that the research was conducted in the absence of any commercial or financial relationships that could be construed as a potential conflict of interest.

## Publisher’s note

All claims expressed in this article are solely those of the authors and do not necessarily represent those of their affiliated organizations, or those of the publisher, the editors and the reviewers. Any product that may be evaluated in this article, or claim that may be made by its manufacturer, is not guaranteed or endorsed by the publisher.

## Abbreviations

CI therapy: Constraint induced therapy
CIMT: Constraint induced movement therapy
mCIMT: Modified CIMT
CIAT: Constraint induced aphasia therapy
ILAT: Intensive language-action therapy
ADL: Activities of daily living
QoL: Quality of life
tDCS: Transcranial direct current stimulation
TMS: Transcranial magnetic stimulation
rTMS: Repetitive TMS
MEP: Motor-evoked potential
CST: Corticospinal tract
fMRI: Functional magnetic resonance imaging
BOLD: Blood oxygenation-level-dependent
M-MAT: Multimodality aphasia therapy

## References

[1] Donkor ES. Stroke in the 21(st) century: a snapshot of the burden, epidemiology, and quality of life. Stroke Res Treat. (2018) 2018:3238165. doi: 10.1155/2018/3238165 30598741 PMC6288566

[2] Tsao CW Aday AW Almarzooq ZI Anderson CAM Arora P Avery CL. Heart disease and stroke statistics-2023 update: a report from the American Heart Association. Circulation. (2023) 147:e93–e621. doi: 10.1161/CIR.0000000000001123, PMID: 36695182 PMC12135016

[3] Cramer SC Richards LG Bernhardt J Duncan P. Cognitive deficits after stroke. Stroke. (2023) 54:5–9. doi: 10.1161/STROKEAHA.122.041775 36542073

[4] Park JG. Update on stroke rehabilitation for non-motor impairment. Brain Neurorehabil. (2022) 15:e13. doi: 10.12786/bn.2022.15.e13, PMID: 36743206 PMC9833475

[5] Kim YW. Update on stroke rehabilitation in motor impairment. Brain Neurorehabil. (2022) 15:e12. doi: 10.12786/bn.2022.15.e12, PMID: 36743199 PMC9833472

[6] Numata K Murayama T Takasugi J Oga M. Effect of modified constraint-induced movement therapy on lower extremity hemiplegia due to a higher-motor area lesion. Brain Inj. (2008) 22:898–904. doi: 10.1080/02699050802425436, PMID: 18850348

[7] Pulvermüller F Neininger B Elbert T Mohr B Rockstroh B Koebbel P. Constraint-induced therapy of chronic aphasia after stroke. Stroke. (2001) 32:1621–6. doi: 10.1161/01.STR.32.7.1621 11441210

[8] Taub E Wolf SL. Constraint induced movement techniques to facilitate upper extremity use in stroke patients. Top Stroke Rehabil. (1997) 3:38–61. doi: 10.1080/10749357.1997.11754128, PMID: 27620374

[9] Morris DM Crago JE DeLuca SC Pidikiti RD Taub E. Constraint-induced movement therapy for motor recovery after stroke. NeuroRehabilitation. (1997) 9:29–43. doi: 10.3233/NRE-1997-9104, PMID: 24526089

[10] Uswatte G Taub E. Constraint-induced movement therapy: a method for harnessing neuroplasticity to treat motor disorders. Prog Brain Res. (2013) 207:379–401. doi: 10.1016/B978-0-444-63327-9.00015-1 24309263

[11] Dreyer FR Doppelbauer L Büscher V Arndt V Stahl B Lucchese G. Increased recruitment of domain-general neural networks in language processing following intensive language-action therapy: fMRI evidence from people with chronic aphasia. Am J Speech Lang Pathol. (2021) 30:455–65. doi: 10.1044/2020_AJSLP-19-00150, PMID: 32830988 PMC7613191

[12] Han P Zhang W Kang L Ma Y Fu L Jia L. Clinical evidence of exercise benefits for stroke. Adv Exp Med Biol. (2017) 1000:131–51. doi: 10.1007/978-981-10-4304-8_9 29098620

[13] Morris DM Taub E Mark VW. Constraint-induced movement therapy: characterizing the intervention protocol. Eura Medicophys. (2006) 42:257–68. PMID: 17039224

[14] Dos Anjos S Morris D Taub E. Constraint-induced movement therapy for lower extremity function: describing the LE-CIMT protocol. Phys Ther. (2020) 100:698–707. doi: 10.1093/ptj/pzz191, PMID: 31899495

[15] Wang D Xiang J He Y Yuan M Dong L Ye Z. The mechanism and clinical application of constraint-induced movement therapy in stroke rehabilitation. Front Behav Neurosci. (2022) 16:828599. doi: 10.3389/fnbeh.2022.828599, PMID: 35801093 PMC9253547

[16] Mark VW Taub E Morris DM. Neuroplasticity and constraint-induced movement therapy. Eura Medicophys. (2006) 42:269–84. PMID: 17039225

[17] Taub E Uswatte G King DK Morris D Crago JE Chatterjee A. A placebo-controlled trial of constraint-induced movement therapy for upper extremity after stroke. Stroke. (2006) 37:1045–9. doi: 10.1161/01.STR.0000206463.66461.97, PMID: 16514097

[18] Alaca N Öcal NM. Proprioceptive based training or modified constraint-induced movement therapy on upper extremity motor functions in chronic stroke patients: a randomized controlled study. NeuroRehabilitation. (2022) 51:271–82. doi: 10.3233/NRE-220009, PMID: 35599504

[19] Fleet A Page SJ MacKay-Lyons M Boe SG. Modified constraint-induced movement therapy for upper extremity recovery post stroke: what is the evidence? Top Stroke Rehabil. (2014) 21:319–31. doi: 10.1310/tsr2104-319, PMID: 25150664

[20] Ju Y Yoon IJ. The effects of modified constraint-induced movement therapy and mirror therapy on upper extremity function and its influence on activities of daily living. J Phys Ther Sci. (2018) 30:77–81. doi: 10.1589/jpts.30.77, PMID: 29410571 PMC5788780

[21] Haddad MM Uswatte G Taub E Barghi A Mark VW. Relation of depressive symptoms to outcome of CI movement therapy after stroke. Rehabil Psychol. (2017) 62:509–15. doi: 10.1037/rep0000171, PMID: 29265871 PMC5745035

[22] Kwakkel G Winters C van Wegen EE Nijland RH van Kuijk AA Visser-Meily A. Effects of unilateral upper limb training in two distinct prognostic groups early after stroke: the explicit-stroke randomized clinical trial. Neurorehabil Neural Repair. (2016) 30:804–16. doi: 10.1177/1545968315624784, PMID: 26747128

[23] Rocha LSO Gama GCB Rocha RSB Rocha LB Dias CP Santos LLS. Constraint induced movement therapy increases functionality and quality of life after stroke. J Stroke Cerebrovasc Dis. (2021) 30:105774. doi: 10.1016/j.jstrokecerebrovasdis.2021.105774, PMID: 33848906

[24] Kim JH Chang MY. Effects of modified constraint-induced movement therapy on upper extremity function and occupational performance of stroke patients. J Phys Ther Sci. (2018) 30:1092–4. doi: 10.1589/jpts.30.1092, PMID: 30154606 PMC6110232

[25] Yadav RK Sharma R Borah D Kothari SY. Efficacy of modified constraint induced movement therapy in the treatment of hemiparetic upper limb in stroke patients: a randomized controlled trial. J Clin Diagn Res. (2016) 10:Yc01–yc5. doi: 10.7860/JCDR/2016/23468.8899 PMC5198445 28050492

[26] El-Helow MR Zamzam ML Fathalla MM El-Badawy MA El Nahhas N El-Nabil LM. Efficacy of modified constraint-induced movement therapy in acute stroke. Eur J Phys Rehabil Med. (2015) 51:371–9. PMID: 25030204

[27] Wang D Li L Pan H Huang L Sun X He C. Comparison of the effects of constraint-induced movement therapy and unconstraint exercise on oxidative stress and limb function-a study on human patients and rats with cerebral infarction. Brain Sci. (2022) 13:4. doi: 10.3390/brainsci13010004, PMID: 36671986 PMC9856592

[28] Mushtaq W Hamdani N Noohu MM Raghavan S. Effect of modified constrain induced movement therapy on fatigue and motor performance in sub acute stroke. J Stroke Cerebrovasc Dis. (2020) 29:105378. doi: 10.1016/j.jstrokecerebrovasdis.2020.105378, PMID: 33080562

[29] Baldwin CR Harry AJ Power LJ Pope KL Harding KE. Modified constraint-induced movement therapy is a feasible and potentially useful addition to the community rehabilitation tool kit after stroke: a pilot randomised control trial. Aust Occup Ther J. (2018) 65:503–11. doi: 10.1111/1440-1630.12488, PMID: 29920688

[30] Ji EK Lee SH. Effects of virtual reality training with modified constraint-induced movement therapy on upper extremity function in acute stage stroke: a preliminary study. J Phys Ther Sci. (2016) 28:3168–72. doi: 10.1589/jpts.28.3168, PMID: 27942143 PMC5140823

[31] Nasb M Li Z Say A Dayoub L Chen H. Comparison of the effects of modified constraint-induced movement therapy and intensive conventional therapy with a botulinum-a toxin injection on upper limb motor function recovery in patients with stroke. Libyan J Med. (2019) 14:1609304. doi: 10.1080/19932820.2019.1609304 31032717 PMC6493286

[32] Seok H Lee SY Kim J Yeo J Kang H. Can short-term constraint-induced movement therapy combined with visual biofeedback training improve hemiplegic upper limb function of subacute stroke patients? Ann Rehabil Med. (2016) 40:998–1009. doi: 10.5535/arm.2016.40.6.998, PMID: 28119829 PMC5256315

[33] Yoon JA Koo BI Shin MJ Shin YB Ko HY Shin YI. Effect of constraint-induced movement therapy and mirror therapy for patients with subacute stroke. Ann Rehabil Med. (2014) 38:458–66. doi: 10.5535/arm.2014.38.4.458, PMID: 25229024 PMC4163585

[34] Bang DH. Effect of modified constraint-induced movement therapy combined with auditory feedback for trunk control on upper extremity in subacute stroke patients with moderate impairment: randomized controlled pilot trial. J Stroke Cerebrovasc Dis. (2016) 25:1606–12. doi: 10.1016/j.jstrokecerebrovasdis.2016.03.030, PMID: 27062417

[35] Bang DH Shin WS Choi HS. Effects of modified constraint-induced movement therapy with trunk restraint in early stroke patients: a single-blinded, randomized, controlled, pilot trial. NeuroRehabilitation. (2018) 42:29–35. doi: 10.3233/NRE-172176, PMID: 29400671

[36] Bang DH Shin WS Choi SJ. The effects of modified constraint-induced movement therapy combined with trunk restraint in subacute stroke: a double-blinded randomized controlled trial. Clin Rehabil. (2015) 29:561–9. doi: 10.1177/0269215514552034, PMID: 25246609

[37] Bang DH Shin WS Choi HS. Effects of modified constraint-induced movement therapy combined with trunk restraint in chronic stroke: a double-blinded randomized controlled pilot trial. NeuroRehabilitation. (2015) 37:131–7. doi: 10.3233/NRE-151245, PMID: 26409698

[38] Lima RC Michaelsen SM Nascimento LR Polese JC Pereira ND Teixeira-Salmela LF. Addition of trunk restraint to home-based modified constraint-induced movement therapy does not bring additional benefits in chronic stroke individuals with mild and moderate upper limb impairments: a pilot randomized controlled trial. NeuroRehabilitation. (2014) 35:391–404. doi: 10.3233/NRE-141130 25227543

[39] Doussoulin A Arancibia M Saiz J Silva A Luengo M Salazar AP. Recovering functional independence after a stroke through modified constraint-induced therapy. NeuroRehabilitation. (2017) 40:243–9. doi: 10.3233/NRE-161409, PMID: 28222546

[40] Doussoulin A Rivas C Rivas R Saiz J. Effects of modified constraint-induced movement therapy in the recovery of upper extremity function affected by a stroke: a single-blind randomized parallel trial-comparing group versus individual intervention. Int J Rehabil Res. (2018) 41:35–40. doi: 10.1097/MRR.0000000000000257, PMID: 28957983

[41] Galvão FRO Silvestre MCA Gomes CLA Pereira NKF Nóbrega VTB Lima WSJ. Group-based constraint-induced movement therapy in the rehabilitation of chronic poststroke patients. Medicine. (2021) 100:e24864. doi: 10.1097/MD.0000000000024864, PMID: 33663110 PMC7909176

[42] Garrido MM Álvarez EE Acevedo PF Moyano VÁ Castillo NN Cavada CG. Early transcranial direct current stimulation with modified constraint-induced movement therapy for motor and functional upper limb recovery in hospitalized patients with stroke: a randomized, multicentre, double-blind, clinical trial. Brain Stimul. (2022) 16:40–7. doi: 10.1016/j.brs.2022.12.008 36584748

[43] Figlewski K Blicher JU Mortensen J Severinsen KE Nielsen JF Andersen H. Transcranial direct current stimulation potentiates improvements in functional ability in patients with chronic stroke receiving constraint-induced movement therapy. Stroke. (2017) 48:229–32. doi: 10.1161/STROKEAHA.116.014988, PMID: 27899754

[44] Kim SH. Effects of dual transcranial direct current stimulation and modified constraint-induced movement therapy to improve upper-limb function after stroke: a double-blinded, pilot randomized controlled trial. J Stroke Cerebrovasc Dis. (2021) 30:105928. doi: 10.1016/j.jstrokecerebrovasdis.2021.105928, PMID: 34256199

[45] Andrade SM Batista LM Nogueira LL de Oliveira EA de Carvalho AG Lima SS. Constraint-induced movement therapy combined with transcranial direct current stimulation over premotor cortex improves motor function in severe stroke: a pilot randomized controlled trial. Rehabil Res Pract. (2017) 2017:1–9. doi: 10.1155/2017/6842549 PMC5303863 28250992

[46] Takebayashi T Takahashi K Moriwaki M Sakamoto T Domen K. Improvement of upper extremity deficit after constraint-induced movement therapy combined with and without preconditioning stimulation using dual-hemisphere transcranial direct current stimulation and peripheral neuromuscular stimulation in chronic stroke patients: a pilot randomized controlled trial. Front Neurol. (2017) 8:568. doi: 10.3389/fneur.2017.00568 29163334 PMC5670104

[47] Abo M Kakuda W Momosaki R Harashima H Kojima M Watanabe S. Randomized, multicenter, comparative study of NEURO versus CIMT in poststroke patients with upper limb hemiparesis: the NEURO-VERIFY study. Int J Stroke. (2014) 9:607–12. doi: 10.1111/ijs.12100, PMID: 24015934

[48] Terranova TT Simis M Santos ACA Alfieri FM Imamura M Fregni F. Robot-assisted therapy and constraint-induced movement therapy for motor recovery in stroke: results from a randomized clinical trial. Front Neurorobot. (2021) 15:684019. doi: 10.3389/fnbot.2021.684019, PMID: 34366819 PMC8335542

[49] Hsieh YW Liing RJ Lin KC Wu CY Liou TH Lin JC. Sequencing bilateral robot-assisted arm therapy and constraint-induced therapy improves reach to press and trunk kinematics in patients with stroke. J Neuroeng Rehabil. (2016) 13:31. doi: 10.1186/s12984-016-0138-5, PMID: 27000446 PMC4802889

[50] Hsieh YW Lin KC Horng YS Wu CY Wu TC Ku FL. Sequential combination of robot-assisted therapy and constraint-induced therapy in stroke rehabilitation: a randomized controlled trial. J Neurol. (2014) 261:1037–45. doi: 10.1007/s00415-014-7345-4, PMID: 24748465

[51] Carrico C Chelette KC 2nd Westgate PM Salmon-Powell E Nichols L Sawaki L. Randomized trial of peripheral nerve stimulation to enhance modified constraint-induced therapy after stroke. Am J Phys Med Rehabil. (2016) 95:397–406. doi: 10.1097/PHM.0000000000000476, PMID: 26945226 PMC4873453

[52] Liu KP Balderi K Leung TL Yue AS Lam NC Cheung JT. A randomized controlled trial of self-regulated modified constraint-induced movement therapy in sub-acute stroke patients. Eur J Neurol. (2016) 23:1351–60. doi: 10.1111/ene.13037, PMID: 27194393

[53] Yingying P Zang L Wang X Yang X. Effect of continuous care combined with constraint-induced movement therapy based on a continuing care health platform on MBI and FMA scores of acute stroke patients. J Healthc Eng. (2022) 2022:1–7. doi: 10.1155/2022/5299969 PMC8808191 35126928

[54] Sawaki L Butler AJ Leng X Wassenaar PA Mohammad YM Blanton S. Differential patterns of cortical reorganization following constraint-induced movement therapy during early and late period after stroke: a preliminary study. NeuroRehabilitation. (2014) 35:415–26. doi: 10.3233/NRE-141132, PMID: 25227542 PMC4484865

[55] Stock R Thrane G Anke A Gjone R Askim T. Early versus late-applied constraint-induced movement therapy: a multisite, randomized controlled trial with a 12-month follow-up. Physiotherapy Res Int. (2018) 23:e1689. doi: 10.1002/pri.1689, PMID: 28686338

[56] Smith MA Tomita MR. Combined effects of telehealth and modified constraint-induced movement therapy for individuals with chronic hemiparesis. Int J Telerehabil. (2020) 12:51–62. doi: 10.5195/ijt.2020.6300, PMID: 32983368 PMC7502810

[57] Gauthier LV Nichols-Larsen DS Uswatte G Strahl N Simeo M Proffitt R. Video game rehabilitation for outpatient stroke (VIGoROUS): a multi-site randomized controlled trial of in-home, self-managed, upper-extremity therapy. EClinicalMedicine. (2022) 43:101239. doi: 10.1016/j.eclinm.2021.101239, PMID: 34977516 PMC8688168

[58] Borstad AL Crawfis R Phillips K Lowes LP Maung D McPherson R. In-home delivery of constraint-induced movement therapy via virtual reality gaming. J Patient Cent Res Rev. (2018) 5:6–17. doi: 10.17294/2330-0698.1550, PMID: 31413992 PMC6664341

[59] Uswatte G Taub E Lum P Brennan D Barman J Bowman MH. Tele-rehabilitation of upper-extremity hemiparesis after stroke: proof-of-concept randomized controlled trial of in-home constraint-induced movement therapy. Restor Neurol Neurosci. (2021) 39:303–18. doi: 10.3233/RNN-201100 34459426

[60] Barzel A Ketels G Stark A Tetzlaff B Daubmann A Wegscheider K. Home-based constraint-induced movement therapy for patients with upper limb dysfunction after stroke (HOMECIMT): a cluster-randomised, controlled trial. Lancet Neurol. (2015) 14:893–902. doi: 10.1016/S1474-4422(15)00147-7, PMID: 26231624

[61] Kelly KM Borstad AL Kline D Gauthier LV. Improved quality of life following constraint-induced movement therapy is associated with gains in arm use, but not motor improvement. Top Stroke Rehabil. (2018) 25:467–74. doi: 10.1080/10749357.2018.1481605, PMID: 30246613 PMC6359892

[62] Rickards T Sterling C Taub E Perkins-Hu C Gauthier L Graham M. Diffusion tensor imaging study of the response to constraint-induced movement therapy of children with hemiparetic cerebral palsy and adults with chronic stroke. Arch Phys Med Rehabil. (2014) 95:506–14.e1. doi: 10.1016/j.apmr.2013.08.245, PMID: 24055785

[63] Sterr A Dean PJ Szameitat AJ Conforto AB Shen S. Corticospinal tract integrity and lesion volume play different roles in chronic hemiparesis and its improvement through motor practice. Neurorehabil Neural Repair. (2014) 28:335–43. doi: 10.1177/1545968313510972, PMID: 24334657

[64] Demers M Varghese R Winstein C. Retrospective analysis of task-specific effects on brain activity after stroke: a pilot study. Front Hum Neurosci. (2022) 16:871239. doi: 10.3389/fnhum.2022.871239, PMID: 35721357 PMC9201099

[65] Lima RC Nascimento LR Michaelsen SM Polese JC Pereira ND Teixeira-Salmela LF. Influences of hand dominance on the maintenance of benefits after home-based modified constraint-induced movement therapy in individuals with stroke. Braz J Phys Ther. (2014) 18:435–44. doi: 10.1590/bjpt-rbf.2014.0050, PMID: 25372006 PMC4228629

[66] Vidal AC Banca P Pascoal AG Santo GC Sargento-Freitas J Gouveia A. Bilateral versus ipsilesional cortico-subcortical activity patterns in stroke show hemispheric dependence. Int J Stroke. (2017) 12:71–83. doi: 10.1177/1747493016672087, PMID: 28004991

[67] Yu C Wang W Zhang Y Wang Y Hou W Liu S. The effects of modified constraint-induced movement therapy in acute subcortical cerebral infarction. Front Hum Neurosci. (2017) 11:265. doi: 10.3389/fnhum.2017.00265 28572764 PMC5435756

[68] Di Lazzaro V Dileone M Capone F Pellegrino G Ranieri F Musumeci G. Immediate and late modulation of interhemipheric imbalance with bilateral transcranial direct current stimulation in acute stroke. Brain Stimul. (2014) 7:841–8. doi: 10.1016/j.brs.2014.10.001, PMID: 25458712

[69] Hu J Li C Hua Y Zhang B Gao BY Liu PL. Constrained-induced movement therapy promotes motor function recovery by enhancing the remodeling of ipsilesional corticospinal tract in rats after stroke. Brain Res. (2019) 1708:27–35. doi: 10.1016/j.brainres.2018.11.011, PMID: 30471245

[70] Abdullahi A Aliyu NU Useh U Abba MA Akindele MO Truijen S. Comparing two different modes of task practice during lower limb constraint-induced movement therapy in people with stroke: a randomized clinical trial. Neural Plast. (2021) 2021:1–9. doi: 10.1155/2021/6664058 PMC7870299 33603778

[71] Duarte Pereira N Ilha J Dos Anjos SM Morris D. Constraint-induced movement therapy for lower extremity use in activities of daily living in people with chronic hemiparesis: multiple case study. Int J Rehabil Res. (2022) 45:215–22. doi: 10.1097/MRR.0000000000000531, PMID: 35634706

[72] Aloraini SM. Effects of constraint-induced movement therapy for the lower extremity among individuals post-stroke: a randomized controlled clinical trial. NeuroRehabilitation. (2022) 51:421–31. doi: 10.3233/NRE-220139, PMID: 35964211

[73] Park SH Hsu CJ Dee W Roth EJ Rymer WZ Wu M. Enhanced error facilitates motor learning in weight shift and increases use of the paretic leg during walking at chronic stage after stroke. Exp Brain Res. (2021) 239:3327–41. doi: 10.1007/s00221-021-06202-9, PMID: 34477919 PMC8541925

[74] Park SH Hsu CJ Dee W Roth EJ Rymer WZ Wu M. Gradual adaptation to pelvis perturbation during walking reinforces motor learning of weight shift toward the paretic side in individuals post-stroke. Exp Brain Res. (2021) 239:1701–13. doi: 10.1007/s00221-021-06092-x, PMID: 33779790 PMC8496127

[75] Park SH Lin JT Dee W Hsu CJ Roth EJ Rymer WZ. Targeted pelvic constraint force induces enhanced use of the paretic leg during walking in persons post-stroke. IEEE Trans Neural Syst Rehabilt Eng. (2020) 28:2184–93. doi: 10.1109/TNSRE.2020.3018397, PMID: 32816677 PMC7652375

[76] Bonnyaud C Zory R Boudarham J Pradon D Bensmail D Roche N. Effect of a robotic restraint gait training versus robotic conventional gait training on gait parameters in stroke patients. Exp Brain Res. (2014) 232:31–42. doi: 10.1007/s00221-013-3717-8, PMID: 24212255

[77] Choi HS Shin WS Bang DH Choi SJ. Effects of game-based constraint-induced movement therapy on balance in patients with stroke: a single-blind randomized controlled trial. Am J Phys Med Rehabil. (2017) 96:184–90. doi: 10.1097/PHM.0000000000000567, PMID: 27386814

[78] Wu M Hsu CJ Kim J. Forced use of paretic leg induced by constraining the non-paretic leg leads to motor learning in individuals post-stroke. Exp Brain Res. (2019) 237:2691–703. doi: 10.1007/s00221-019-05624-w, PMID: 31407027 PMC6755123

[79] Hsu CJ Kim J Roth EJ Rymer WZ Wu M. Forced use of the paretic leg induced by a constraint force applied to the nonparetic leg in individuals poststroke during walking. Neurorehabil Neural Repair. (2017) 31:1042–52. doi: 10.1177/1545968317740972, PMID: 29145773 PMC6083449

[80] Rose ML Nickels L Copland D Togher L Godecke E Meinzer M. Results of the COMPARE trial of constraint-induced or multimodality aphasia therapy compared with usual care in chronic post-stroke aphasia. J Neurol Neurosurg Psychiatry. (2022) 93:573–81. doi: 10.1136/jnnp-2021-328422, PMID: 35396340 PMC9148985

[81] Pulvermüller F Berthier ML. Aphasia therapy on a neuroscience basis. Aphasiology. (2008) 22:563–99. doi: 10.1080/02687030701612213, PMID: 18923644 PMC2557073

[82] Woldag H Voigt N Bley M Hummelsheim H. Constraint-induced aphasia therapy in the acute stage: what is the key factor for efficacy? A randomized controlled study. Neurorehabil Neural Repair. (2017) 31:72–80. doi: 10.1177/1545968316662707, PMID: 27506677

[83] Mozeiko J Myers EB Coelho CA. Treatment response to a double administration of constraint-induced language therapy in chronic aphasia. J Speech Lang Hear Res. (2018) 61:1664–90. doi: 10.1044/2018_JSLHR-L-16-0102, PMID: 29872835 PMC8645245

[84] Doppelbauer L Mohr B Dreyer FR Stahl B Büscher V Pulvermüller F. Long-term stability of short-term intensive language-action therapy in chronic aphasia: a 1-2 year follow-up study. Neurorehabil Neural Repair. (2021) 35:861–70. doi: 10.1177/15459683211029235, PMID: 34232091

[85] Szaflarski JP Ball AL Vannest J Dietz AR Allendorfer JB Martin AN. Constraint-induced aphasia therapy for treatment of chronic post-stroke aphasia: a randomized, blinded, controlled pilot trial. Med Sci Monit. (2015) 21:2861–9. doi: 10.12659/MSM.894291, PMID: 26399192 PMC4588672

[86] Stahl B Millrose S Denzler P Lucchese G Jacobi F Flöel A. Intensive social interaction for treatment of poststroke depression in subacute aphasia: the CONNECT trial. Stroke. (2022) 53:3530–7. doi: 10.1161/STROKEAHA.122.039995, PMID: 36124755

[87] Stahl B Mohr B Dreyer FR Lucchese G Pulvermüller F. Using language for social interaction: communication mechanisms promote recovery from chronic non-fluent aphasia. Cortex. (2016) 85:90–9. doi: 10.1016/j.cortex.2016.09.021, PMID: 27842269

[88] Stahl B Mohr B Büscher V Dreyer FR Lucchese G Pulvermüller F. Efficacy of intensive aphasia therapy in patients with chronic stroke: a randomised controlled trial. J Neurol Neurosurg Psychiatry. (2018) 89:586–92. doi: 10.1136/jnnp-2017-315962, PMID: 29273692 PMC6031278

[89] Heikkinen PH Pulvermüller F Mäkelä JP Ilmoniemi RJ Lioumis P Kujala T. Combining rTMS with intensive language-action therapy in chronic aphasia: a randomized controlled trial. Front Neurosci. (2018) 12:1036. doi: 10.3389/fnins.2018.01036 30778280 PMC6369187

[90] Martin PI Treglia E Naeser MA Ho MD Baker EH Martin EG. Language improvements after TMS plus modified CILT: pilot, open-protocol study with two, chronic nonfluent aphasia cases. Restor Neurol Neurosci. (2014) 32:483–505. doi: 10.3233/RNN-130365, PMID: 25015701 PMC4592134

[91] Kavian S Khatoonabadi AR Ansari NN Saadati M Shaygannejad V. A single-subject study to examine the effects of constrained-induced aphasia therapy on naming deficit. Int J Prev Med. (2014) 5:782–6. PMID: 25013699 PMC4085932

[92] Szaflarski JP Griffis J Vannest J Allendorfer JB Nenert R Amara AW. A feasibility study of combined intermittent theta burst stimulation and modified constraint-induced aphasia therapy in chronic post-stroke aphasia. Restor Neurol Neurosci. (2018) 36:503–18. doi: 10.3233/RNN-180812, PMID: 29889086

[93] Allendorfer JB Nenert R Nair S Vannest J Szaflarski JP. Functional magnetic resonance imaging of language following constraint-induced aphasia therapy primed with intermittent Theta burst stimulation in 13 patients with post-stroke aphasia. Med Sci Monit. (2021) 27:e930100. doi: 10.12659/MSM.930100 33970893 PMC8120906

[94] Mohr B Difrancesco S Harrington K Evans S Pulvermüller F. Changes of right-hemispheric activation after constraint-induced, intensive language action therapy in chronic aphasia: fMRI evidence from auditory semantic processing. Front Hum Neurosci. (2014) 8:919. doi: 10.3389/fnhum.2014.00919 25452721 PMC4231973

[95] Nenert R Allendorfer JB Martin AM Banks C Ball A Vannest J. Neuroimaging correlates of post-stroke aphasia rehabilitation in a pilot randomized trial of constraint-induced aphasia therapy. Med Sci Monit. (2017) 23:3489–507. doi: 10.12659/MSM.902301, PMID: 28719572 PMC5529460

[96] Mohr B MacGregor LJ Difrancesco S Harrington K Pulvermüller F Shtyrov Y. Hemispheric contributions to language reorganisation: an MEG study of neuroplasticity in chronic post stroke aphasia. Neuropsychologia. (2016) 93:413–24. doi: 10.1016/j.neuropsychologia.2016.04.006, PMID: 27063061

[97] Taub E Mark VW Uswatte G. Implications of CI therapy for visual deficit training. Front Integr Neurosci. (2014) 8:78. doi: 10.3389/fnint.2014.00078 25346665 PMC4191165

[98] Pereira DD Eras-Garcia R Frange C de Oliveira CB Tufik S Coelho FMS. The influence of sleep quality and circadian preferences on upper extremity rehabilitation in stroke patients after constraint-induced movement therapy. Int J Rehabil Res. (2020) 43:20–7. doi: 10.1097/MRR.0000000000000379, PMID: 31688240

[99] Lee HJ Moon HI Kim JS Yi TI. Is there a dose-dependent effect of modified constraint-induced movement therapy in patients with hemiplegia? NeuroRehabilitation. (2019) 45:57–66. doi: 10.3233/NRE-192721, PMID: 31403953

[100] Thrane G Askim T Stock R Indredavik B Gjone R Erichsen A. Efficacy of constraint-induced movement therapy in early stroke rehabilitation: a randomized controlled multisite trial. Neurorehabil Neural Repair. (2015) 29:517–25. doi: 10.1177/1545968314558599, PMID: 25398726

